# Urinary Neuroendocrine Neoplasms Treated in the “Modern Era”: A Multicenter Retrospective Review

**DOI:** 10.1016/j.clgc.2023.02.009

**Published:** 2023-03-17

**Authors:** Bryan Khuong Le, Patrick McGarrah, Alan Paciorek, Amr Mohamed, Andrea B. Apolo, David L. Chan, Diane Reidy-Lagunes, Haley Hauser, Jaydira Del Rivero, Julia Whitman, Kathleen Batty, Li Zhang, Nitya Raj, Tiffany Le, Emily Bergsland, Thorvardur R. Halfdanarson

**Affiliations:** a Department of Medicine, University of California, San Francisco, CA; b Division of Medical Oncology, Mayo Clinic, Rochester, MN; c Department of Medicine, Memorial Sloan-Kettering Cancer Center, New York, New York, USA; d Center for Cancer Research, National Cancer Institute, National Institutes of Health, Bethesda, Maryland, USA; e UH Seidman Cancer Center, Case Western Reserve University, Cleveland, OH; f Department of Medical Oncology, Royal North Shore Hospital, St. Leonards, NSW 2065, Sydney, New South Wales, Australia; g Vanderbilt University; h Department of Epidemiology and Biostatistics, University of California, San Francisco, CA

**Keywords:** Modern era treatment, Poorly differentiated bladder neuroendocrine carcinoma, Poorly differentiated urinary neuroendocrine carcinoma, Well-differentiated renal neuroendocrine tumors, Well-differentiated urinary neuroendocrine tumor

## Abstract

**Background:**

Primary urinary neuroendocrine neoplasms (U-NENs) are extremely rare thus optimal treatment is unknown. Grading and treatment are typically extrapolated from other primary sites. Since 2010, the clinical landscape for NENs has changed substantially. We performed a retrospective review of U-NENs to assess treatment patterns and oncologic outcomes of patients treated in the recent era of NEN therapy.

**Patients and Methods:**

A multicenter retrospective review of patients diagnosed after 2005 and alive after 2010. Time to treatment failure (TTF) was used to evaluate progression and toxicity for systemic therapy. Tumors were categorized as having either well-differentiated neuroendocrine tumor (WDNET) or poorly differentiated neuroendocrine carcinoma (PDNEC) histology.

**Results:**

A total of 134 patients from 6 centers were included in our analysis, including 94 (70%) bladder, 32 (24%) kidney, 2 (1.5%) urethra and 4 other urinary primaries (3.0%). Poorly-differentiated neuroendocrine carcinoma was more common in bladder (92%) than non-bladder tumors (8%). Median Ki-67 available in bladder primary was 90% (n = 24), kidney 10% (n = 23), ureter 95% (n = 1), urethra 54% (n = 2), and others 90% (n = 3). Patients received a median of 2 therapies (range 0–10). Median time to death was not reached in locoregional WDNETs versus 8.2 years (95% CI, 3.5-noncalculable) in metastatic WDNETs (predominantly renal primary). Median time to death was 3.6 years (95% CI, 2.2–9.2) in locoregional PDNECs versus 1 year (95% CI, 0.8–1.3) in metastatic PDNECs (predominantly bladder primary).

**Conclusion:**

This is the most extensive series examining treatment patterns in patients with U-NENs in the recent era of NEN therapy. The apparent inferior survival for bladder NENs is likely due to the preponderance of PDNECs in this group. As predicted, treatments for U-NENs mirrored that of other more common NENs. In our retrospective cohort, we observed that patients with WD-UNETs treated with peptide receptor radionuclide therapy (PRRT) and everolimus suggested potential activity for disease control in WD-UNETs. Prospective studies are needed to assess the activity of new oncology drugs in UNENs.

## Introduction

Neuroendocrine neoplasms (NENs) are a rare and heterogeneous group of tumors that arise from neuroendocrine cells in nearly all organs in the body. The most frequent sites are the gastrointestinal (GI) tract (74%) and lungs (25%), with the genitourinary system being an uncommon site of origin (*<*1%).^1–3^ Although these malignancies are rare, the incidence and prevalence of NENs are steadily increasing. A large study using data from the Surveillance, Epidemiology, and End Results (SEER) program showed a nearly 7-fold increase in age-adjusted NEN incidence from 1973 (1.09 per 100,000) to 2012 (6.98 per 100,000) in the United States.^4^ Heterogeneity with respect to site of origin, grading and staging criteria, and frequent revisions to histopathologic classification schemes present challenges to studying NENs.

Nevertheless, several common themes help guide clinical management and define prognosis. Regardless of the primary site, it is essential to determine stage and differentiation.^5^ Well-differentiated neuroendocrine tumors (NETs) and poorly differentiated carcinomas (PDNECs) typically demonstrate vastly divergent clinical behavior and are genetically different diseases often with TP53 and Rb mutations.^3,4,6^ In gastro-entero-pancreatic (GEP) NENs, the histologic grade determined using the Ki-67 proliferation index or mitotic rate also plays a central role in predicting clinical behavior. Treatment decisions depend on multiple factors, including tumor stage, differentiation, grade, clinical behavior, and extent and bulk of disease.^7^ Typically, advanced disease is incurable.

Primary urinary neuroendocrine neoplasms (U-NENs) are extremely rare and comprise only 1% to 2% of all genitourinary (GU) malignancies.^2,8,9^ Published data consist largely of case reports and small series focused on primaries of a single organ such as the kidney.^10^ U-NENs are known to arise in the kidney, bladder, renal pelvis, and ureter.^11,12^ U-NENs of the bladder account for an estimated *<* 1% of all primary bladder cancers,^13,14^ and only about 100 cases of renal NENs have been reported since 1966.^11^ The 2016 WHO (World Health Organization) classification of tumors of the Urinary System and Male Genital Organs classifies NENs of the kidney, prostate, and bladder into well-differentiated neuroendocrine tumors (WDNETs), poorly differentiated neuroendocrine carcinomas (PDNECs) (small cell neuroendocrine carcinoma (SCNEC) and large cell neuroendocrine carcinoma (LCNEC)), and paragangliomas.^15^

Currently, there is no established grading system for U-NENs based on mitotic rate or Ki-67 proliferation as in GEP and lung NENs. As with the more common NEN primary sites, WD-UNETs are thought to have better survival outcomes than PD-UNECs, which demonstrate much more aggressive behavior and frequent metastases. Current treatment strategies for U-NENs are ill-defined and extrapolated from GEP and lung NENs,^10^ as site-specific guidelines are limited or do not currently exist for this uncommon group of malignancies.^16^ Previously published studies are based on literature reviews combining retrospective data from multiple institutions; thus long term follow-up data are lacking, with minimal detail regarding clinicopathologic features or treatment patterns. Additionally, U-NENs are not often included in NEN clinical trials given their rarity, limiting rigorous analysis of available or new treatments.

Following the WHO 2010 grading criteria for GEP-NENs (incorporating the routine use of Ki-67 proliferation index or mitotic index for grading), several FDA-approved treatments emerged for GEP and lung NENs, advancing the treatment landscape for patients with these malignancies. Treatments employed include resection for early-stage disease, somatostatin analogs (approved in GEP-NETs), ^177^Lu-DOTA-octreotate peptide receptor radionuclide therapy (PRRT) (approved in GEP-NETs), liver-directed therapies (eg, hepatic artery embolization and thermal ablation), targeted agents such as everolimus (approved in GEP-NETs and lung NETs) and sunitinib (approved in pancreatic NETs) and systemic chemotherapies for unresectable NETs (eg, temozolomide-based therapy for pancreatic NETs). Platinum-based cytotoxic chemotherapy continues to be the standard of care for PDNECs of any sites (with or without resection and/or radiation depending on the stage).^17^ Front-line chemoimmunotherapy is now approved for extensive-stage SCLC.^18,19^ Combination immunotherapy is now listed as a reasonable second-line option in NCCN NEN guidelines for high grade disease based on data from nonrandomized studies in extrapulmonary NENs.^17,20,21^

Per NCCN guidelines for bladder cancers, any small cell component (or neuroendocrine features) with localized invasive disease, should be treated with neoadjuvant chemotherapy (with cisplatin/etoposide or carboplatin/etoposide), followed by radical cystectomy or definitive chemoradiotherapy^16,22^; Immune checkpoint blockade combined with cytotoxic chemotherapy has yielded long-term remissions in some patients with metastatic small cell lung cancer,^18^ but the precise role of immunotherapy remains unclear in UNENs.

To examine treatment pattern of U-NENs in this “modern” era of therapy for NENs of other sites, we performed a multicenter retrospective review to assess treatment patterns of U-NENs patients treated in the era of contemporary guidelines (post-2010).

## Methods

### Patients, Eligibility Criteria and Data Collection

This study was approved by the UCSF Committee for Human Research (IRB number 10–00854). Researchers at each participating site identified patients from their institutions who had previously been diagnosed with NEN of the urinary tract. Eligible primary sites included the bladder, kidney, urethra, and ureter. Mixed tumors and tumors of any stage were allowed. Paragangliomas and primary tumors of the reproductive system were excluded. Primary prostatic NEN was excluded, given that it is an already well-recognized entity with substantial published data regarding tumor characteristics and treatments.^23–25^ All cases were diagnosed after 2005 and had undergone internal pathology review at the participating sites. Patients had to be alive after 2010 to be included. Demographic, clinical, and pathologic data were abstracted from the medical record, including race/ethnicity, smoking status, location of the tumor, tumor grade, differentiation, extent of disease at diagnosis (metastatic vs. non-metastatic), treatment history, and survival.

### Statistical Analyses

**Figure 1:**
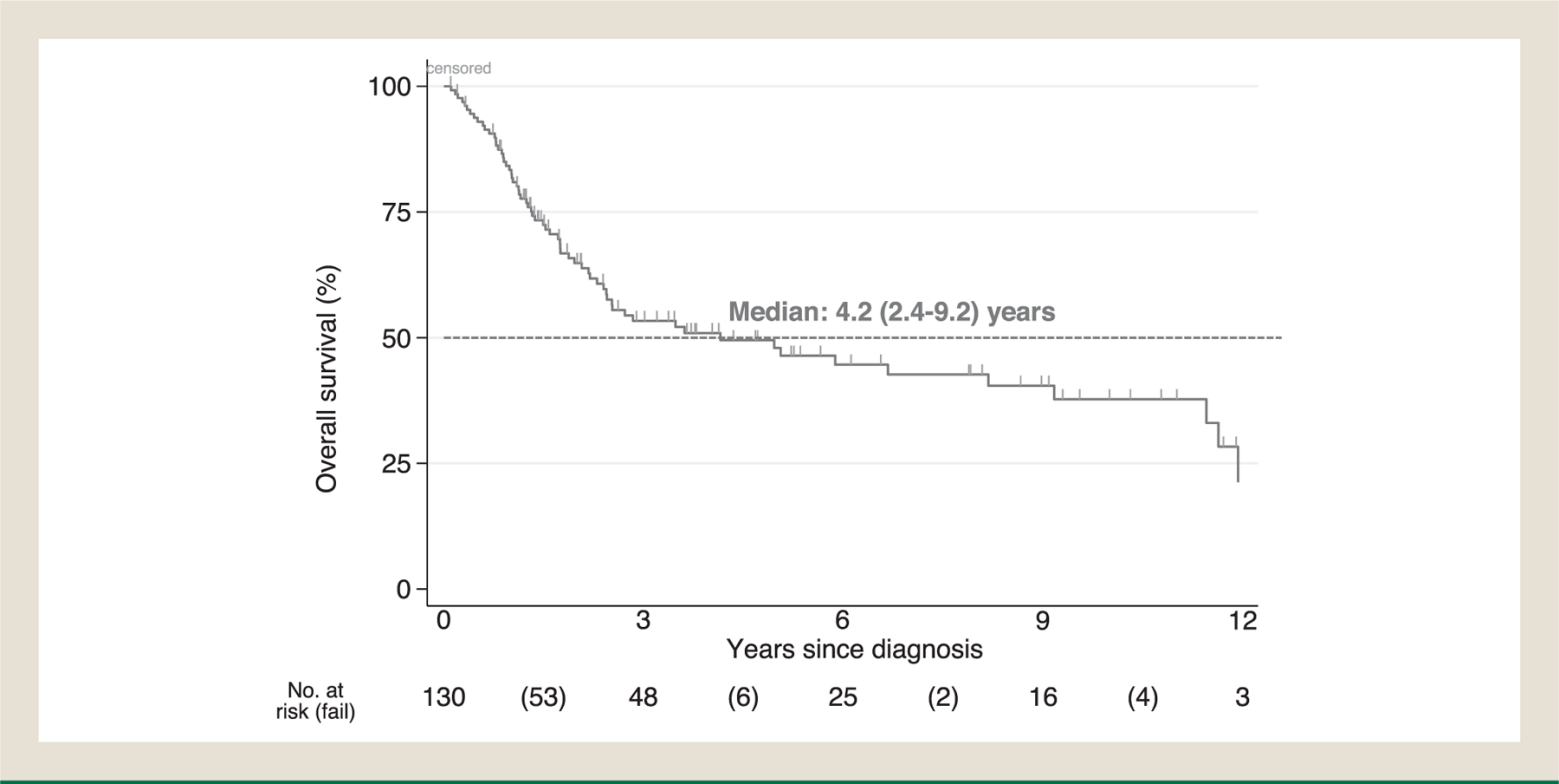
Kaplan-Meier estimate of overall survival in total cohort (N = 130^∗^). ^∗^4 pts excluded due to unknown survival status. Median OS in cohort: 4.2 years.

**Figure 2:**
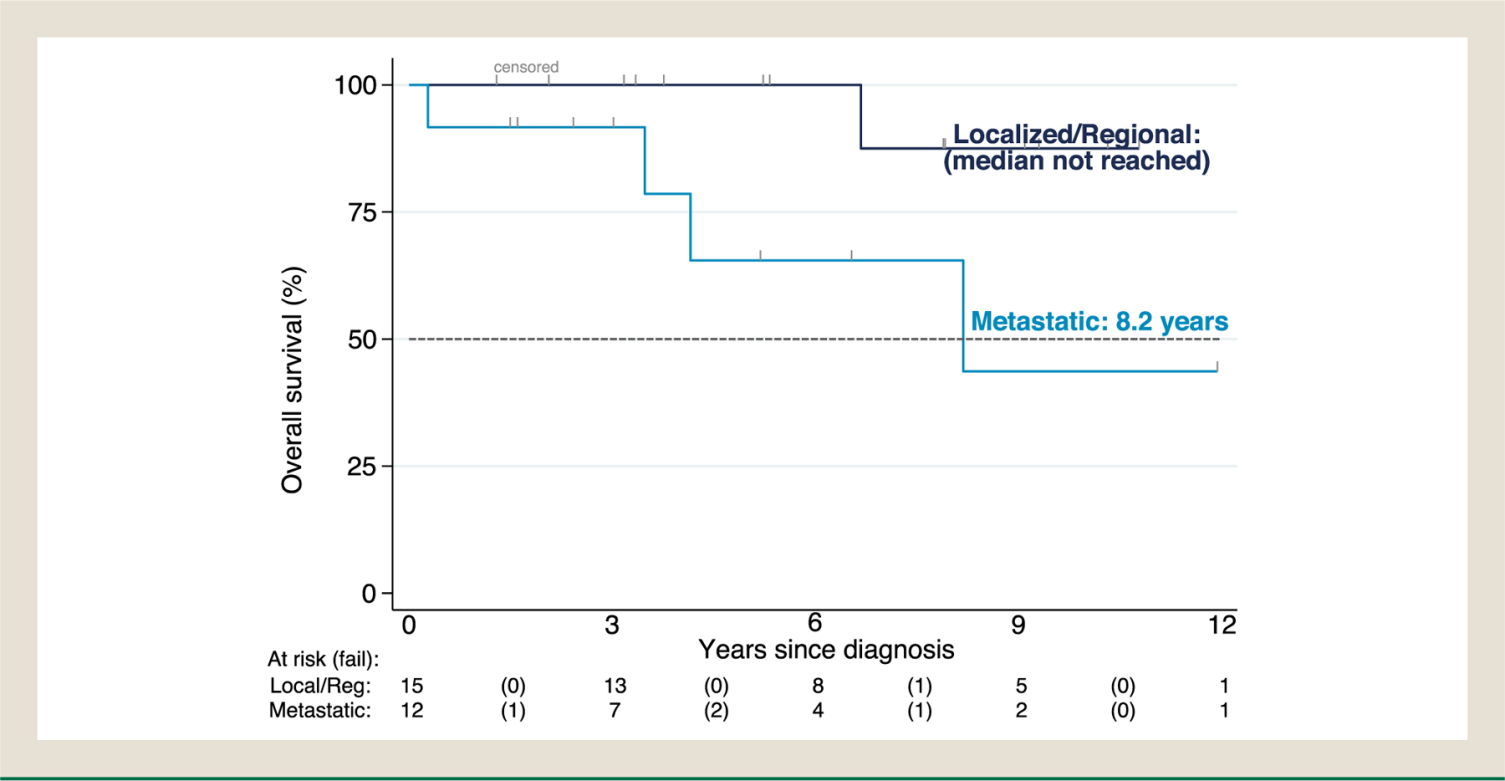
Kaplan-Meier estimate of overall survival in WDNETs by staging at diagnosis (N = 27). Median OS in localized/regional disease: not reached; metastatic 8.2 years (95% CI, 3.5- noncalculable).

**Figure 3:**
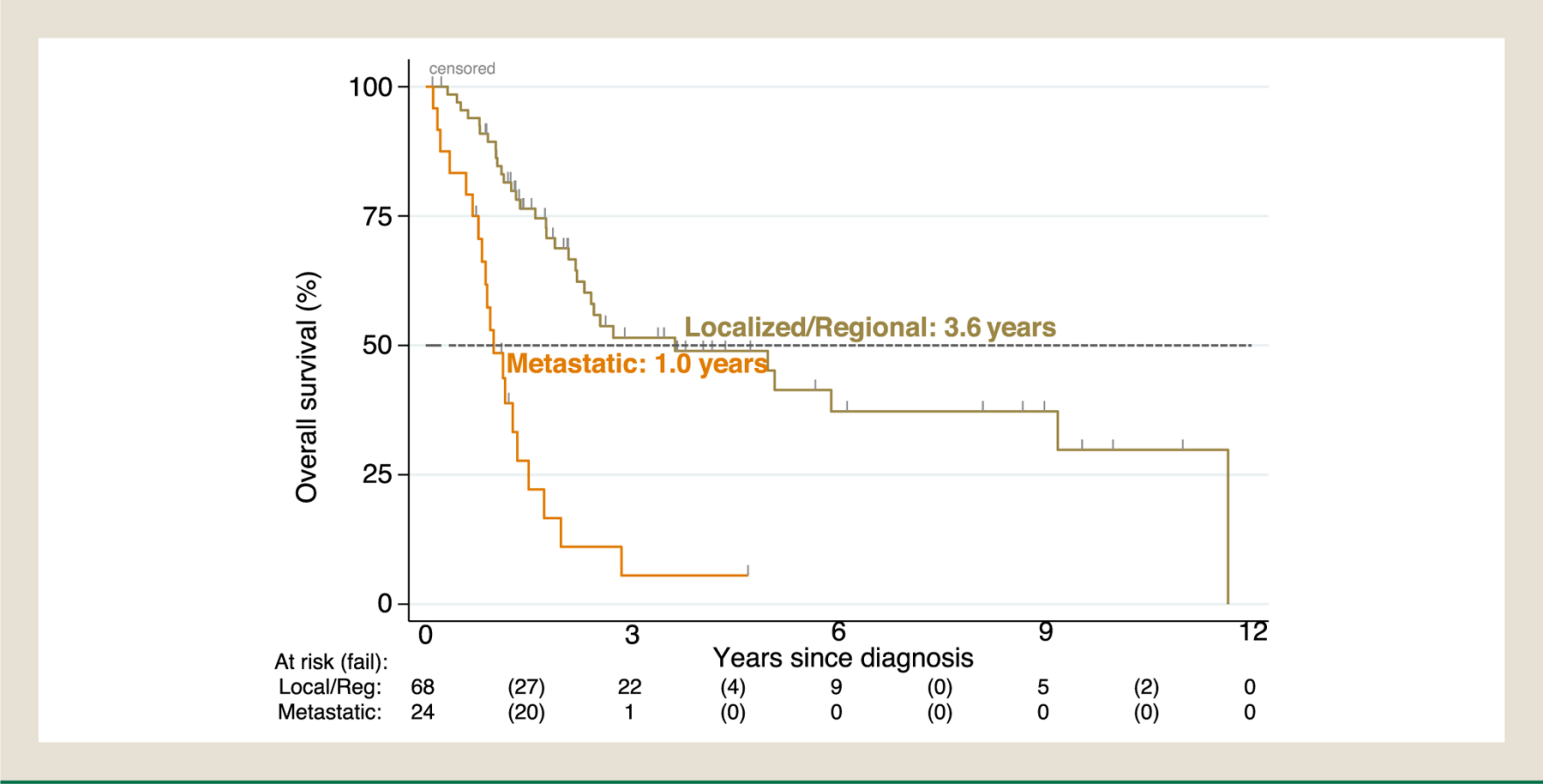
Kaplan-Meier estimate of overall survival in PDNECs by staging at diagnosis (N = 92^∗^). ^∗^3 pts excluded due to unknown survival status. Median OS in localized/regional disease: 3.6 years (95% CI, 2.2–9.2); metastatic: 1 year (95% CI, 0.8–1.3).

Patients’ demographic, clinical characteristics and survival outcomes were summarized by descriptive statistics. Specifically, median with range was used to describe continuous variables, frequency with percentage was used to summarize categorical variables, and Kaplan-Meier was used to summarize overall survival. Efficacy of treatments was assessed using a proxy endpoint, time to treatment failure (TTF), as formal RECIST measurements were not performed. TTF was measured in months for oral targeted agents, capecitabine/temozolomide, PRRT and immunotherapy, defined as the time from initiation to discontinuation (months) of such treatment due to documented progression determined by the treating physician, toxicity, or death, whichever came first. Platinum-based therapy and SSA were not accounted for median TTF as patients received in neoadjuvant/adjuvant setting (platinum-based) or concomitantly with other therapies throughout treatment course (SSA). In addition to TTF, for the purpose of constructing the swimmer plots visualizing treatment changes (treatment efficacy or tolerability was not assessed), time to next treatment (TTNT) was captured for localized/focal treatments such as surgery, liver-directed therapy, radiation, platinum-based therapy and SSA, defined as the interval from commencement of one treatment to initiation of the next line of therapy. Treatments were classified, sequenced, and counted, then reported in tables of frequencies. Sankey diagrams were constructed to show treatment sequences throughout disease continuum by tumor differentiation status (WDNET, PDNEC) and staging.^26^ Overall survival (OS) was defined as the time from the date of pathologic diagnosis to the date of death of any cause or last follow up. Survival was summarized for various patient subgroups using Kaplan-Meier curves (Figures 1–3) or medians (Table 4), but was not formally tested for differences because the scope of our study focuses on describing the clinical course of these patients but not predicting outcomes. Analyses were performed using STATA (StataCorp), R (R foundation for statistical computing), SEER^∗^Stat software, and Joinpoint Regression Program.

## Results

### Clinico-Pathologic Characteristics

Our study included 6 participating sites with a total of 134 patients diagnosed between 2005 and 2020 and alive after 2010. The baseline demographic and clinicopathologic characteristics in the total cohort are reported in Table 1. The median age at diagnosis was 63 years. In our series, 30% of patients were female (n = 40) and 21% were identified as nonwhite (n = 28) or unreported (n = 3). Caucasian males were over-represented in bladder primary group (predominantly PDNECs); sex predilection was not observed among patients with other primaries. The primary tumor sites were bladder (n = 94, 70.1%), kidney (n = 32, 23.9%), ureter (n = 2, 1.5%), urethra (n = 2, 1.5%) or unspecified urinary primary sites (n = 4, 3.0%). At the time of analysis, 53 (40%) patients were alive, 65 (48.5%) patients were deceased, and 16 (11.9%) were lost to follow-up.

Histologic grade extrapolated from WHO classifications for GEP-NENs showed the following distribution in our cohort: grade 1 (n = 9, 6.7%), grade 2 (n = 18, 13.4%), grade 3 (n = 99, 73.9%), and unknown (n = 8, 6.0%). According to the pathology records, 27 patients (20.2%) had well-differentiated NETs, 95 (70.9%) had poorly differentiated NECs, and 12 (9.0%) had NENs for which differentiation was not reported (NR). PDNECs consisted of 84 (88.4%) with small cell morphology, 6 (6.3%) with large cell morphology, 4 (4.2%) with ambiguous morphology and 1 (1.1%) unknown. Grade 3 WDNET in GEP-NENs, defined as morphologically well-differentiated NET with a Ki-67 *>* 20%, has been recognized in WHO NEN classification schemes since 2017 (pancreas) and 2019 (gastrointestinal).^27^ Two of 27 WDNET patients in our series had tumors with Ki-67 of 25% and 30%, raising the possibility of grade 3 WDNETs in UNENs.

Forty-one patients were reported to have metastatic disease at diagnosis, including 10 of 32 (31%) kidney and 25 of 94 (27%) bladder primaries. FDG or Dotatate avidity information was based on imaging reports at participating institutions. Out of 69 PDNEC patients with 18F-FDG PET/CT imaging, 94% demonstrated FDG-avid (n = 65; 63 bladder, 2 kidney, 1 ureter, 1 urethra, 2 others). 68Ga-Dotatate-PET/CT imaging was performed in 18 patients with WDNETs, 89% (n = 16, 15 kidney, 1 urethra) were reported to have Dotatate-avid and 75% (6/8) reported to have FDG-avid. Two renal WDNETs (Ki-67 index of 10% and 15% respectively) showed avidity on 18F-FDG PET/CT and 68Ga-Dotatate-PET/CT. Interestingly, horseshoe kidney was present in 5 patients (16%) with renal primary WDNET in our series.

### Treatment Modalities

In our series, the predominant treatments for early-stage PDNECs were surgery plus chemotherapy 32% (21/65) and surgery alone 26% (17/65) (Table 2). In stage IV disease, platinum-based therapy 61% (23/38) and immune checkpoint inhibitors 34% (13/38) were the most common systemic treatments (Table 3). Most stage IV PDNEC patients underwent platinum-based therapy as the first systemic treatment 8 of 38 (21%) (Table 5).

For WD-UNETs, all (16/16, 100%) early-stage patients in our cohort underwent surgery upfront. 19 of 21 (90%) patients with metastatic WDNETs underwent resection of primary tumor at some point during the disease course. SSAs were the most common systemic therapy (15/21, 71%) for metastatic WD-UNETs, followed by oral targeted agents 11 of 21 (52%) (Table 3). Most stage IV patients underwent treatment with SSAs 10 of 21 (48%) or primary tumor resection 8 of 21 (38%) as their first treatment. The majority of patients (18/21, 86%) received second-line therapy, with 5 of 18 (28%) receiving oral targeted agents and 3 of 18 (17%) undergoing liver-directed therapy. Patients also received SSA in subsequent lines, but the proportion of other therapies increased in later lines, including targeted agents, capecitabine/temozolomide, and ^177^Lu-Dotatate PRRT. Of note, patients in our cohort received PRRT later in the disease course, starting at fourth or fifth-line treatment (Table 5). The Sankey diagrams depict the therapies that each patient received and demonstrate the complexity of treatment patterns in each patient’s disease course (Supplemental Figures 3–5). A total of 10 patients with Dotatate-avid, stage IV disease in our cohort underwent treatment with PRRT, including one PDNEC bladder primary, one ambiguous differentiation with seminal vesicle primary and 8 WDNETs (7 renal and 1 urethral primaries).

### Patient Outcomes Relative to Clinicopathologic and Treatment Characteristics

The median follow-up varied by tumor sites: bladder primary (n = 91) 18 months, kidney (n = 31), 64 months, ureter (n = 2) 11 months, urethra (n = 2) 34 months, and other sites (n = 4) 16 months (Table 1). The median overall survival in our cohort (n = 130) was 4.2 (95% CI, 2.4–9.2) years (Figure 1). Median time to death was not reached in locoregional WDNETs versus 8.2 years (95% CI, 3.5-noncalculable) in metastatic WDNETs (predominantly renal primary) (Figure 2). Median time to death was 3.6 years (95% CI, 2.2–9.2) in locoregional PDNECs versus 1 year (95% CI, 0.8–1.3) in metastatic PDNECs (predominantly bladder primary) (Figure 3). In the 22 patients with Dotatate-avid (92% of n = 24 tested), the median survival was 12 years (95% CI, 8.2-NC) (Table 4). The survival probability at 10 years for localized WDNET patients is 88% (95% CI 39–98) and for metastatic WDNET is 44% (95% CI 8–77) (Figure 2). The survival probability at 3 years for localized PDNEC patients is 52% (95% CI 37–64) and for metastatic PDNEC is 6% (95% CI 1–22) (Figure 3). Other characteristics associated with estimated OS are included in Table 4.

## Discussion

U-NENs are extremely rare, and published data are limited to small case series and case reports.^28–30^ Available population-level data suggests that the incidence of NENs across all sites is rising, including NENs of urinary origin.^8,13^

This is the first large-scale assessment of treatment patterns in U-NENs in the era of modern NEN treatment guidelines and based on clinicopathologic features. Available guidance regarding the overall prognosis and optimal care for U-NENs is limited; current treatment recommendations are extrapolated from studies performed in other more common NENs. To our knowledge, this is the only series on U-NENs that describes treatment patterns and descriptive OS for patients treated in the “modern era” of GEP-NEN and lung NEN therapy. Most of our patients had either kidney (n = 32) or bladder (n = 94) U-NEN primary, which led to a natural comparison between these 2 groups. The majority of patients with kidney primary had WDNETs histology 25 of 32 (78%), while most tumors from the bladder were PDNECs 87 of 94 (93%). Patients with bladder NENs had overall poorer outcomes, with shorter survival times than patients with kidney NEN primary, likely owing to tumors of bladder origin more often being PDNECs, even though they were less likely to be metastatic at diagnosis.

### Poorly Differentiated Bladder NEC

The most common treatment for early-stage PD-UNECs in our cohort was surgery alone or surgery with other treatment modalities, including chemotherapy or chemoradiation (Table 2). NCCN guidelines for bladder cancer recommend neoadjuvant chemotherapy followed by radical cystectomy or definitive chemoradiotherapy as consolidation for any small cell component with localized disease.^16^ For metastatic disease, the most common systemic treatments were platinum-based therapy up-front, followed by maintenance immunotherapy and immunotherapy in the salvage setting lines or upfront immunotherapy in platinum-ineligible patients. Platinum-based chemotherapy was the most common systemic therapy for NECs in our cohort, consistent with current NCCN guidelines for bladder cancer and other studies.^31–34^ In addition to platinum-based therapy, alternating ifosfamide plus doxorubicin with etoposide plus cisplatin regimen has been described specifically for small cell carcinoma of the bladder.^35^ As PDNECs encompass a spectrum of Ki-67 index values, from 20% to 100%, the response rate of platinum-based therapy in UNECs might vary depending on Ki-67 percentage, as retrospective data suggest that in advanced high-grade GI NECs, tumors with Ki-67 21% to 55% are less likely to respond to platinum-based chemotherapy than tumors with Ki-67 *>*55% (15% v 42% response rate, *P <* .001).^36^ In our cohort of U-PDNECs (primarily of bladder origin), the median Ki-67 was 90% but ranged from 20% to 100%. The efficacy of second-line therapy and beyond for extrapulmonary PDNECs is poor, and no standard treatment has emerged as superior.^37^ In GEP PDNECs, there is no standard salvage therapy, with second-line regimens are often extrapolated from refractory small cell lung cancer ≤ 6 months as are organ-specific chemotherapy regimens for non NENs of the site of origin.^38^ Temozolomide-based chemotherapy may be active in platinum-refractory disease with approximately 20% to 40% response rate from retrospective studies^39,40^; however, randomized prospective data are lacking, and the data are mainly for large cell NEC.

The role of immune checkpoint inhibitor therapy is under study in NENs. A small portion of our metastatic patients 16% (6/38) had immunotherapy as first-line systemic therapy. In our cohort, immunotherapy was primarily received at third and fourth-line systemic therapy for metastatic PDNECs (Table 5). Single-agent-check point inhibitor (CPI) was the most common immunotherapy received in metastatic U-PDNECs (n = 8), with a limited median TTF of 2 months (Table 3). Previous studies have shown single-agent anti-PD1 and anti-PD-L1 drugs seem to have little activity in patients with extrapulmonary NECs.^41–44^ However, single agent-CPI in combination with chemotherapy showed mixed results in GEP-NENs and SCLC. In EP-NECs, a phase II trial pembrolizumab + chemotherapy (paclitaxel or irinotecan) has shown limited activity based on preliminary data with overall response rate of 9%, median progression-free survival (PFS) of 2 months and median OS of 4 months.^45^ In SCLC, CASPIAN demonstrated a statistically significant and clinically meaningful improvement OS in durvalumab plus platinum/etoposide versus platinum/etoposide alone in extensive stage SCLC.^19^ Phase III trial of atezolizumab, carboplatin, and etoposide in SCLC showed improved OS and slightly improved PFS without improved response rate.^18^ Dual checkpoint inhibition with anti-PD-1/PD-L1 and anti-CTLA-4 antibodies (ipilimumab/nivolumab) may have activity in high-grade extrapulmonary NENs.^20,46,47^ For example, in 1 study, 8 of 18 high-grade NENs of different primary sites (including lung) showed a response rate of 44% versus 0 in low and intermediate-grade NET.^46^ However, a follow-up study revealed 26% response rate in high grade NENs specifically^20^ and a single-center retrospective study showed very modest activity of the dual checkpoint inhibition in patients with refractory NECs.^48^ It is unclear whether these data apply to urinary NEC or if immunotherapy plays a role in the post-platinum setting of bladder NECs. Clinical trials are currently the preferred option in a refractory setting if available.

### Well-Differentiated Renal NET

We observed that all patients 16 of 16 (100%) with early-stage WD-UNETs underwent primary tumor resection. 19 (90%) stage IV patients also commonly underwent primary tumor resection at some point during their disease course (Table 3). Nephrectomy was the primary treatment choice for locoregional WD-UNET, and SSA 71% (15/21) was the most common systemic treatment choice for metastatic WDNET renal primary in our series. The precise role of aggressive upfront surgical resection (including lymphadenectomy and hepatic metastasectomy) is unclear given the lack of data from randomized trials, but some series have described positive outcomes.^10,49,50^ As there are no consensus guidelines for systemic therapy in renal NENs, patients in our cohort received treatments extrapolated from GEP WDNET. SSAs are often employed either to control hormone-mediated symptoms and/or for disease control in WD-GEPNETs.^51,52^ Therefore, it seems reasonable to consider SSA in patients with metastatic WD-UNETs with favorable biology and with evidence of somatostatin receptor expression (eg, Dotatate avidity on PET imaging).

Other common systemic therapies for stage IV WDNETs in our cohort were everolimus, with median TTF of 9.3 months (n = 8) and PRRT with median TTF of 9.6 months ranging from 3 to 17 months (N = 7), suggesting potential activity in WD-UNENs. Ongoing follow-up for 2 patients still responding to PRRT at the time of data lock, as patients have not all progressed or discontinued treatment due to toxicity. The swimmer plots show the variety in timing of the many treatments that our patients received (Supplemental Figures 1 and 2). TKIs have a well-established place in therapy in renal cell carcinoma^53–55^; other kinase inhibitors may have a role for tumor control, such as cabozantinib, pazopanib, surufatinib, axitinib, and lenvatinib based on results in well-differentiated GEP-NETs,^56–60^ but efficacy data regarding WD-UNETs specifically are lacking. PRRT is now FDA-approved in somatostatin receptor-expressing GEP-NET based on improved PFS in mid-gut NET with ^177^Lu-Dotatate compared to high dose octreotide.^61^ Until more data become available, it is reasonable to consider the use of everolimus or PRRT for patients with metastatic well-differentiated urinary NETs, with PRRT should be limited to patients with somatostatin receptor-positive imaging. Temozolomide-based therapy (n = 6) was associated with a median TTF of 3 months (range 2–40 months) (Table 3). A previous prospective trial in PNET showed that capecitabine/temozolomide is superior to temozolomide monotherapy in low-grade PNET, with median PFS of 22.7 months versus 14.4 months (HR = 0.58, *P* = .023).^62^ Another multicenter retrospective study showed a median time to treatment failure of 5.7 months in WD G3 GEP-NETs.^39^ Ultimately, the efficacy of temozolomide in U-NENs remains unclear from our analysis.

In our series, horseshoe kidney was present in 5 WD renal primary (16%). Previous reports showed that NENs associated with horseshoe kidneys have indolent clinical behavior.^63^ Better outcomes have also been reported for patients with tumors arising from horseshoe kidneys in the literature^12,64,65^).

### Strengths and Limitations

Our approach is limited by the retrospective nature of the study. There were several important variables with incomplete data for some subjects, including differentiation, grade, and total time on treatment. This may have skewed the results toward over or under-estimating the importance of certain predictor variables relative to overall survival. Another limitation is the lack of centralized pathology review, but all participating centers are tertiary centers with substantial experience managing patients with NENs. Results observed in our study are based on the 6 participating centers that may not reflect the real-world practice patterns in other institutions. Recent data in patients with pancreatic NETs have suggested different outcomes among patients treated at different types of facilities, where treatment at an academic center was associated with better outcomes.^66^ We also did not perform any statistical comparisons as our study was intended to be descriptive and given small sample sizes.

Nevertheless, we believe this study is significant because it provides the first comprehensive analysis of the clinical behavior and treatment patterns of U-NENs treated in a modern era of therapy for NENs. Furthermore, we limited eligibility to urinary primaries other than prostate, and excluded paragangliomas and reproductive system primaries that display different tumor biology, to characterize a subgroup of NENs not previously studied. We have also presented data for 2 key U-NEN groups (bladder and kidney primaries), highlighting significant differences between these sites. Perhaps most notably, our study reported outcomes and treatment patterns in U-NENs during recent era therapy for NENs. This is important given the number of FDA-approved and NCCN-recommended therapies that have become available in the last ten years after demonstrating improved outcomes in patients with GEP and lung primaries.^17^

## Conclusion

There is a need for evidence-based guidelines on managing patients with U-NENs. Presently, classification, grading schemes and clinical management are extrapolated from recommendations for more common primary sites of NENs.

In our series, lines of therapy varied widely within and between UNENs arising in different organ sites. The survival of bladder NENs was inferior to non-bladder NEN, likely because the bladder NENs are almost uniformly poorly differentiated. Our median OS for PD-UNECs and WD-UNENs were similar to previous EP-PDNECs of the bladder and WD-UNENs.

We observed that agents commonly used in lung and GEP-NENs are routinely used in U- NENs. Larger (and ideally prospective) series are needed to fully understand the efficacy of PRRT, everolimus and temozolomide-based therapy in U-NETs. Platinum-based therapy was the most common regimen used for bladder NECs. The role of checkpoint inhibitors in PD-UNECs remains unknown. Because prospective series on rare tumors such as U-NENs are lacking, it will be essential to continue systematic assessment of the experiences with U-NENS from large volume NEN centers. Until more data becomes available, it is reasonable for providers to extrapolate data generated in other NEN sites when selecting therapy for UNENs. The current study is the largest of its kind and the first to study U-NENs treated in the recent era of NEN therapy.

## Supplementary Material

1

## Figures and Tables

**Table 1:** Baseline Patient Characteristics, Clinical and Pathologic Findings (n = 134)

Characteristic	Total Patients n (%)	Bladder (n = 94) n (%)	Kidney (n = 32) n (%)	Ureter (n = 2) n (%)	Urethra (n = 2) n (%)	Unknown (n = 4) n (%)
Age at diagnosis (years)						
Median	63	68	49	65	61	55
Range	22–89	25–89	22–78	56–73	53–68	39–84
Time to last follow-up						
Median (months)	25	18	64	11	34	16
N^a^	130	91	31	2	2	4
Demographics						
Sex						
Female	40 (29.9)	21 (22.3)	17 (53.1)	1 (50)	2 (100)	3 (75)
Male	94 (70.2)	73 (77.6)	15 (46.8)	1 (50)	0	1 (25)
Hispanic/Latino						
Yes	9 (6.7)	7 (7.45)	2 (6.3)	0	0	0
No	120 (89.6)	82 (87.2)	30 (93.7)	2 (100)	2 (100)	4 (100)
Unknown	5 (3.7)	5 (5.32)	0	0	0	0
Race						
American Indian/Alaska native	2 (1.49)	0	2 (6.3)	0	0	0
Asian	12 (8.96)	7 (7.5)	4 (12.5))	0	0	1 (25)
Black/African American	4 (2.99)	3 (3.2)	1 (3.13)	0	0	0
Native Hawaiian/Other Pacific Islander	2 (1.49)	0	2 (6.3)	0	0	0
Other Race	8 (5.97)	6 (6.4)	2 (6.3)	0	0	0
White	103 (76.87)	75 (79.8)	21 (65.6)	2 (100)	2 (100)	3 (75)
Not reported	3 (2.24)	3 (3.2)	0	0	0	0
Metastatic at diagnosis						
No	91 (67.9)	69 (73.4)	20 (63)	1 (50)	1 (50)	0
Yes	41 (30.5)	25 (26.6)	10 (31)	1 (50)	1 (50)	4 (100)
Not reported	2 (1.49)	0	2 (6.2)	-	-	-
Differentiation						
Well	27 (20.2)	0	25 (78.1)	0	1 (50)	1 (25)
Poorly	95 (70.9)	87 (92.6)	3 (9.4)	2 (100)	1 (50)	2 (50)
Ambiguous/Not reported	12 (8.9)	7 (7.4)	4 (12.5)	0	0	1 (25)
Grade						
1	9 (6.7)	1 (1.06)	8 (25)	0	0	0
2	18 (13.4)	0	15 (46.9)	1 (50)	1 (50)	1 (25)
3	99 (73.9)	89 (94.7)	5 (15.6)	1(50)	1 (50)	3 (75)
Not reported	8 (6.0)	4 (4.24)	4 (12.5)	0	0	0
Available Ki-67						
Median (N, Range)	70 (53, 1–100)	90 (24, 40–100)	10 (23, 1–90)	95 (1, NA)	54 (2, 13–95)	90 (3, 20–95)
Additional clinical features						
Horseshoe kidney						
No	128 (95.5)	93 (99)	27 (84)	2 (100)	2 (100)	4 (100)
Yes	5 (3.7)	0	5 (16)	0	0	0
Unknown	1 (0.8)	1 (1)	0	0	0	0

a 4 pts was excluded due to unknown survival status at the time of data collection

**Table 2:** Treatment Modalities for Locoregional Urinary NENs at diagnosis (N = 87)

Treatment Regimens	Localized/Regional Disease (N = 87)		
	Well-Differentiated NET (n = 16) N (%)	Poorly Differentiated NEC (n = 65) N (%)	NR/Ambiguous Differentiation (n = 6) N (%)
**Resection of primary tumor**	16 (100%)	58 (84%)	5 (83%)
Surgery alone	16 (100%)	17 (26%)	2 (33%)
Surgery plus chemotherapy	0	21 (32%)	2 (33%)
Surgery plus chemoradiation	0	10 (15%)	1 (17%)
Surgery plus radiation	0	1 (2%)	0
Surgery plus immunotherapy	0	1 (2%)	0
Surgery plus chemotherapy/immunotherapy	0	1 (2%)	0
Surgery plus chemotherapy/radiation	0	3 (5%)	0
Surgery plus chemotherapy/chemoradiation	0	4 (6%)	0
**Primary tumor left in place**	0	7 (11%)	1 (17%)
Chemotherapy alone	N/A	2 (3%)	0
Chemoradiation alone	N/A	2 (3%)	0
Chemotherapy plus radiation	N/A	0	1 (17%)
Chemotherapy plus chemoradiation	N/A	2 (3%)	0
Chemotherapy plus immunotherapy	N/A	1 (1%)	0

4 PDNEC pts who did not receive treatment during localized/regional stage were excluded. Resection of WDNET included nephrectomy. Resection of PDNEC included cystectomy with or without TURBT; (41*/*65 (63%) received TURBT only without cystectomy, 24*/*65 (37%) received cystectomy with/without TURBT). Resection of NR/Ambiguous differentiation included nephrectomy, cystectomy, and TURBT. Chemotherapy included: platinum-based, platinum-based/topoisomerase inhibitors. Chemoradiation included capecitabine, mitomycin c + 5-fluorouracil, platinum-based, taxol, and unknown chemotherapy

**Table 3:** Treatment Modalities for Metastatic^a^ Urinary NENs (N = 67)

Treatment Regimens	Stage IV Disease at Any Time Point (N = 67)				
	Well-Differentiated NET (N = 21)		Poorly Differentiated NEC (N = 38)		Unknown/ambiguous Differentiation (N = 8)
	Patients N (%)	Median TTF^b^ (mo) range, N’	Patients N (%)	Median TTF^b^ (mo) range, N’	Patients N (%)
Resection (primary and/or metastasis)	11 (52%)	-	21 (55%)	-	5 (63%)
Resection of the primary	9 (43%)	-	18 (47%)	-	3 (38%)
Resection of primary at any given time in the disease continuum^a^	19 (90%)	-	31 (79%)	-	6 (75%)
Radiation	5 (24%)	-	8 (21%)	-	3 (38%)
Chemoradiation	0	-	2 (5%)	-	0
Targeted agents (oral)	11 (52%)^c^	-	7 (18%) ^c^	-	2 (25%)
VEGFR	4 (19%)	3 (*<*1–28) N = 7	6 (16%)	3 (*<*1–5) N = 7	1 (13%)
EGFR	1 (5%)	-	0	**-**	0
PARP	0	-	1 (3%)	**-**	0
mTOR (everolimus)	9 (43%)	9.3 (2–21) N = 8	0	**-**	2 (25%)
SSA	15 (71%)	-	2 (5%)	-	3 (38%)
Chemotherapy	7 (33%)^c^	-	23 (61%) ^c^	-	7 (88%) ^c^
Capecitabine/Temozolomide	6 (29%)	3 (2–40) N = 6	2 (5%)	-	3 (38%)
Platinum-based	2 (10%)	-	23 (61%)	-	4 (50%)
Other chemotherapy	2 (10%)	-	7 (18%)	-	4 (50%)
Immunotherapy	2 (10%)	-	13 (34%) ^c^	-	-
Single-agent CPI (atezo, pembro, nivo)	2 (10%)	**-**	10 (26%)	2 (1–3)	
N = 8	0				
Other immunotherapy	0	-	5 (13%)	-	1 (13%)
PRRT	8 (38%)	9.6 (3–17)			
N= 7	1 (3%)	-	1 (13%)		
Liver-directed therapy	8 (38%)	-	0	-	1 (13%)

a Metastatic disease included stage IV disease at diagnosis or patients developed stage IV disease ≥ 6 months from UNEN diagnosis. 21 WDNETs included: 11 stage IV at diagnosis and 10 with localized/regional disease at diagnosis later developed metastatic disease; 38 PDNEC included 24 stage IV at diagnosis and 14 localized/regional disease at diagnosis later developed metastatic disease; 8 Not reported/Unknown differentiation included 5 stage IV at diagnosis and 3 localized/regional disease at diagnosis later developed metastatic disease.

b Available TTFs of systemic therapies with N*>* 5 were reported, except for platinum-based therapy (excluded due to neoadjuvant/adjuvant settings), and SSA (excluded due to some pts received continuously throughout disease course)

c pts received at least 1 or more oral target agents, chemotherapy, or immunotherapy, resulting in counts and percentages that may not sum to the total n or 100%.WDNETs-Primary resection included nephrectomy and urethrectomy. One patient received SSA concurrently with other treatments, including capecitabine/temozolomide, PRRT, and hepatic wedge resection. TTF was available in 7 pts who underwent PRRT, with 2 patients still responding at the time of data collection cut-off timepoint. SSA was used concurrently in 1 primary resection (nephrectomy), 5 everolimus. Capecitabine was used concurrently in 1 PRRT treatment. Other chemotherapy included oxaliplatin and taxane-based therapy.

PDNECs-Resection: Out of 18 pts received primary resection, 16 pts with metastatic disease at diagnosis received primary resection, and 2 pts who had received TURBT during localized disease at diagnosis later received cystectomy at stage IV. 10 (26%) received nephrectomy, ureterectomy, nephroureterectomy, cystoprostatectomy or cystectomy with or without TURBT. Platinum-based therapy included cisplatin or carboplatin with etoposide or cisplatin or carboplatin with gemcitabine. Other chemotherapy included topoisomerase inhibitors, paclitaxel alone or with gemcitabine, and methotrexate/folinic acid. Immunotherapy: 2 pembrolizumab and radiation used concurrently. Other immunotherapy included: durvalumab/tremelimumab, platinum-based/atezolizumab, ipilimumab/nivolumab, cabozantinib/ipilimumab/nivolumab, pembrolizumab/olaparib. Targeted agents: 1 ramucirumab combined with folfiri.

Not reported/ambiguous differentiation: other immunotherapy included avelumab/NHS-IL12. 1 patient received SSA concurrently with other treatments, including everolimus, capecitabine/temozolomide, pazopanib. Other chemotherapy included: gemcitabine/ifosfamide/cisplatin; cisplatin/irinotecan, ifosfamide/doxorubicin, and oxaliplatin.

Abbreviations- atezo = atezolizumab; nivo = nivolumab; pembro = pembrolizumab; SSA = somatostatin analogs; TURBT = transurethral resection of bladder tumor. Targeted agents included VEGFR inhibitors (bevacizumab, cabozantinib, pazopanib, sunitinib); mTOR (everolimus), EGFR inhibitor (erlotinib).

**Table 4:** Overall Survival in Years Since Diagnosis

Characteristic	No. of Patients	No. of Deaths	Median (95% CI)
Age at Diagnosis (Years)			
≤39	11	3	12.0 (6.7-NC)
40–60	38	17	8.2 (2.2-NC)
61–70	36	18	2.5 (1.3-NC)
≥71	37	22	2.4 (1.1–5.0)
Demographics			
Sex			
Female	39	16	6.7 (2.2-NC)
Male	91	49	2.8 (2.1–8.2)
Ethnicity			
Not Hispanic/Latino	121	62	3.6 (2.3–8.2)
Hispanic/Latino	9	3	9.2 (0.3-NC)
Race			
White	100	52	3.6 (2.3–11.5)
Non-white	27	12	5.1 (1.3-NC)
Smoking History			
Never	52	19	11.5 (4.2-NC)
Ever	75	44	2.3 (1.8–5.0)
Diagnostics			
FDG avid (n = 85 tested)			
No	5	1	11.5 (NC)
Yes	74	45	2.4 (1.8–3.6)
DOTA avid (n = 24 tested)			
No	2	1	0.3 (NC)
Yes	22	5	12.0 (8.2-NC)
Pathologic findings			
Primary site			
Kidney	31	6	NC
Ureter	2	2	0.6 (0.6-NC)
Bladder	91	53	2.2 (1.7–2.8)
Urethra	2	1	2.7 (2.7-NC)
Other urinary primary	4	3	1.5 (0.3-NC)
Staging at diagnosis			
Not metastatic at diagnosis	88	37	6.7 (2.7–12.0)
Metastatic at diagnosis	41	28	1.5 (1.0–3.5)
Differentiation			
Well or ambiguous	28	6	NC
Poor	92	53	2.2 (1.7–3.6)
Grade			
1	9	2	NC
2	18	3	NC
3	95	56	2.2 (1.6–2.8)

**Table 5:** Treatment Regimen Sequence in Metastatic^a^ Unens by Differentiation

Treatment Sequence	Well-Differentiated NET N (%)	Poorly Differentiated NEC N (%)	Unknown/ambiguous Differentiation N (%)
Treatment 1	N = 21	N = 38	N = 8
TURBT	0	13 (34%)	0
Platinum-based therapy	2 (10%)	8 (21%)	3 (38%)
SSA	10 (48%)	1 (3%)	0
Resection	8 (38%)	4 (11%)	3 (38%)
Immunotherapy	0	6 (16%)	1 (13%)
Targeted agents	1 (5%)	2 (5%)	0
Treatment 2	N = 18	N = 26	N = 7
Targeted agents	5 (28%)	1 (4%)	0
Liver directed therapy	3 (17%)	0	0
Radiation	2 (11%)	2 (8%)	2 (29%)
Temozolomide ± Capecitabine	2 (11%)	0	0
Resection	1 (6%)	6 (23%)	1 (14%)
Platinum-based therapy	0	11 (42%)	1 (14%)
Immunotherapy	1 (6%)	2 (8%)	0
SSA	2 (11%)	1 (4%)	2 (29%)
Treatment 3	N = 17	N = 14	N = 6
Liver directed therapy	2 (12%)	0	0
Resection	6 (35%)	1 (7%)	1 (17%)
SSA	2 (12%)	0	1 (17%)
Immunotherapy	0	3 (21%)	0
Platinum-based therapy	0	4 (29%)	1 (17%)
Targeted agents	5 (29%)	2 (14%)	1 (17%)
Treatment 4	N = 12	N = 11	N = 4
Immunotherapy	0	3 (27%)	0
Targeted agents	3 (25%)	2 (18%)	1 (25%)
SSA	2 (17%)	0	0
Resection	2 (17%)	0	0
Temozolomide ± Capecitabine	1 (8%)	2 (18%)	2 (50%)
PRRT	2 (17%)	0	0
Treatment 5	N = 9	N = 6	N = 4
Topoisomerase inhibitors	0	2 (33%)	0
Targeted agents	3 (33%)	0	1 (25%)
Temozolomide ± Capecitabine	2 (22%)	0	1 (25%)
Liver directed therapy	1 (11%)	0	0
PRRT	1 (11%)	0	0

a Metastatic disease included stage IV disease at diagnosis or pts developed stage IV disease ≥ 6 months from UNENs diagnosis

## Clinical Practice Points

- Due to its rarity, future prospective studies will require multicenter effort to assess treatment efficacy in UNENs.
- Primary kidney neuroendocrine neoplasms are likely to have well-differentiated histology, while most tumors from the bladder are poorly differentiated neuroendocrine carcinomas.
- It is reasonable to adapt treatments from other common NEN primary sites (gastroenteropancreatic neuroendocrine neoplasms and lung) to treatment of urinary NENs.
- Overall, the survival in urinary NEN is favorable with a multi-year survival. However, bladder primary sites are usually poorly differentiated and have a 1-year median survival.

## Abbreviations:

Anti-CTLA: Cytotoxic T Lymphocyte associated Antigen 4
CI: Confidence Interval
EGFR: Epidermal Growth Factor Receptor
FDA: Food and Drug Administration
FDG PET/CT: Fluorodeoxyglucose Positron Emission Tomography
Ga68 DOTA: Gallium 68 Dotatate
IRB: Institutional Review Board
mTORI: mammalian Target of Rapamycin Inhibitor
NCCN: National Comprehensive Cancer Network
No.: Number
NR: Not Reported
PARPI: Poly ADP-Ribose Polymerase Inhibitors
PD1: Programmed cell Death protein 1
PD-L1: Programmed cell Death Ligand 1
PRRT: Peptide Receptor Radionuclide Therapy
Pts: Patients
RECIST: Response Evaluation Criteria in Solid Tumor
RR: Response Rate
SEER: Surveil-lance, Epidemiology, and End Results
Supp.: Supplemental
TKIs: Tyrosine Kinase inhibitors
TURBT: Trans Urethral Resection of Bladder Tumor
VEGFR: Vascular Endothelial Growth Factor Receptor
