# Supplementary material for: Urinary Neuroendocrine Neoplasms Treated in the “Modern Era”: A Multicenter Retrospective Review
Source: Clin Genitourin Cancer. Author manuscript; Available in PMC 2024 Aug 2. (PMC11296333; doi:10.1016/j.clgc.2023.02.009)

Supplementary material

Supplemental Figure 1 Swimmer's Plots illustrating treatment schema in bladder primary. TTF and TTNT in months used to develop swimmer plots to visualize treatment changes. Patients received at least one of the following treatments were included in swimmer's plots (supp. Figure 1 & supp. Figure 2): PRRT, oxaliplatin, temozolomide +/- capecitabine, immunotherapy, targeted agent, topoisomerase inhibitor, taxane-based chemotherapy. Bladder primary cohort (N=32). Pt who received only platinum-based therapy, surgical interventions, or chemoradiation without the above listed treatments were excluded.

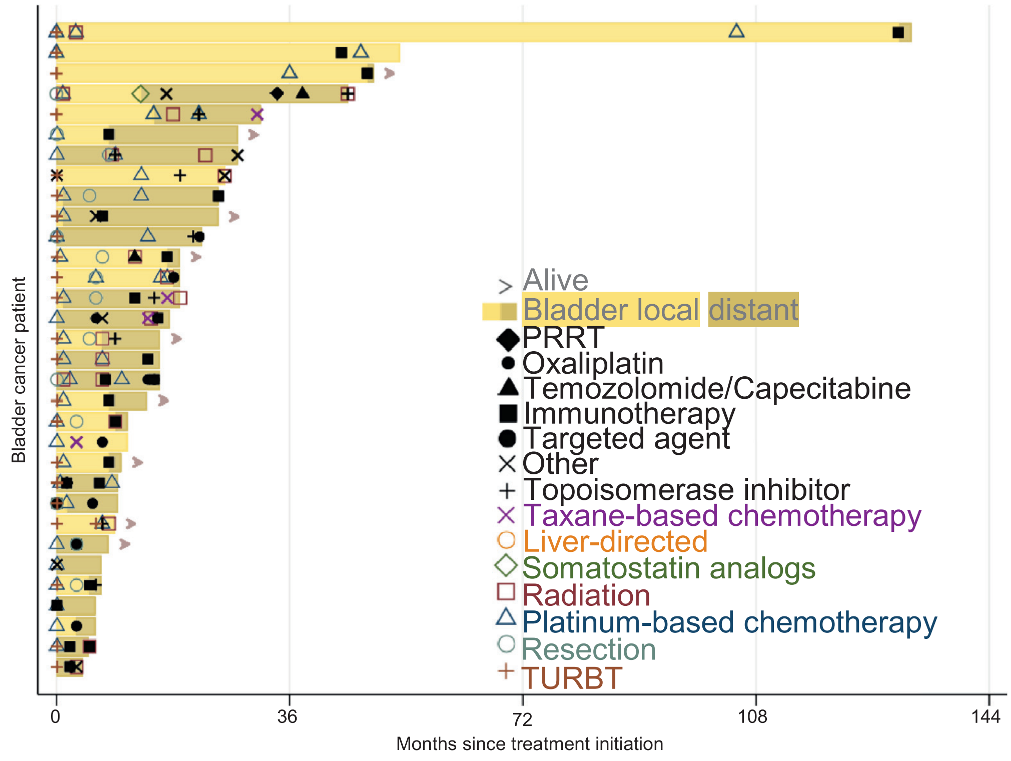

Supplemental Figure 2 Swimmer's Plots illustrating treatment schema in kidney and other non-bladder urinary cohort. TTF and TTNT in months used to develop swimmer plots to visualize treatment changes. Patients having at least one of the following treatments were included in swimmer's plots (supp. Figure 1 & supp. Figure 2): PRRT, oxaliplatin, temozolomide +/- capecitabine, immunotherapy, targeted agent, topoisomerase inhibitor, taxane-based chemotherapy. Kidney and non-bladder urinary primary (N=24). Pt who received SSA only or surgical interventions without the listed treatments were excluded.

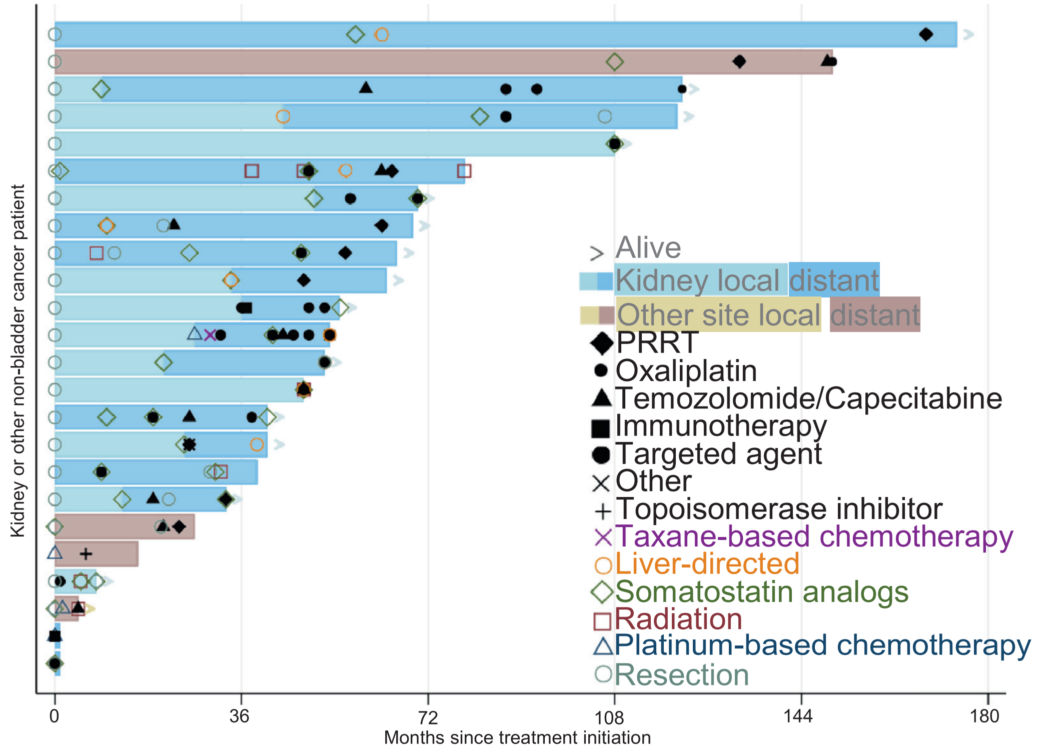

Supplemental Figure 3 Sankey diagrams illustrating treatment sequence from first-line up to tenth-line therapy in PDNECs (N=93). One patient excluded from the treatment sequence due to not reported treatment information. Resection included cystectomy, cystoprostatectomy, nephrectomy, nephroureterectomy, lymphadenectomy, brain dissection. Platinum-based therapy included carboplatin or cisplatin + etoposide. Chemoradiation included capecitabine, platinum-based, mitomycin c + 5-fluorouracil, taxane-based therapy. Immunotherapy included: bacillus calmette-guerin, PD-1/PD-L1 inhibitors +/- anti- CTLA-4 (atezolizumab, pembrolizumab, nivolumab (alone or in combination with Ipilimumab), durvalumab + tremelimumab), PD-1/PD-L1 inhibitors + anti- CTLA-4 + VEGFR (ipilimumab + nivolumab + cabozantinib), PD-1/PD-L1 inhibitors + platinum-based therapy (atezolizumab + cisplatin (or carboplatin)/etoposide). Targeted agents included VEGFR inhibitor (cabozantinib), PARPI (olaparib), VEGFR2 inhibitor (ramucirumab). Others included gemcitabine, ifosfamide + doxorubicin, methotrexate + folinic acid, unknown chemotherapy, platinum-based therapy + topoisomerase inhibitors. Abbreviations: Loc = localized disease; Dis = distant.

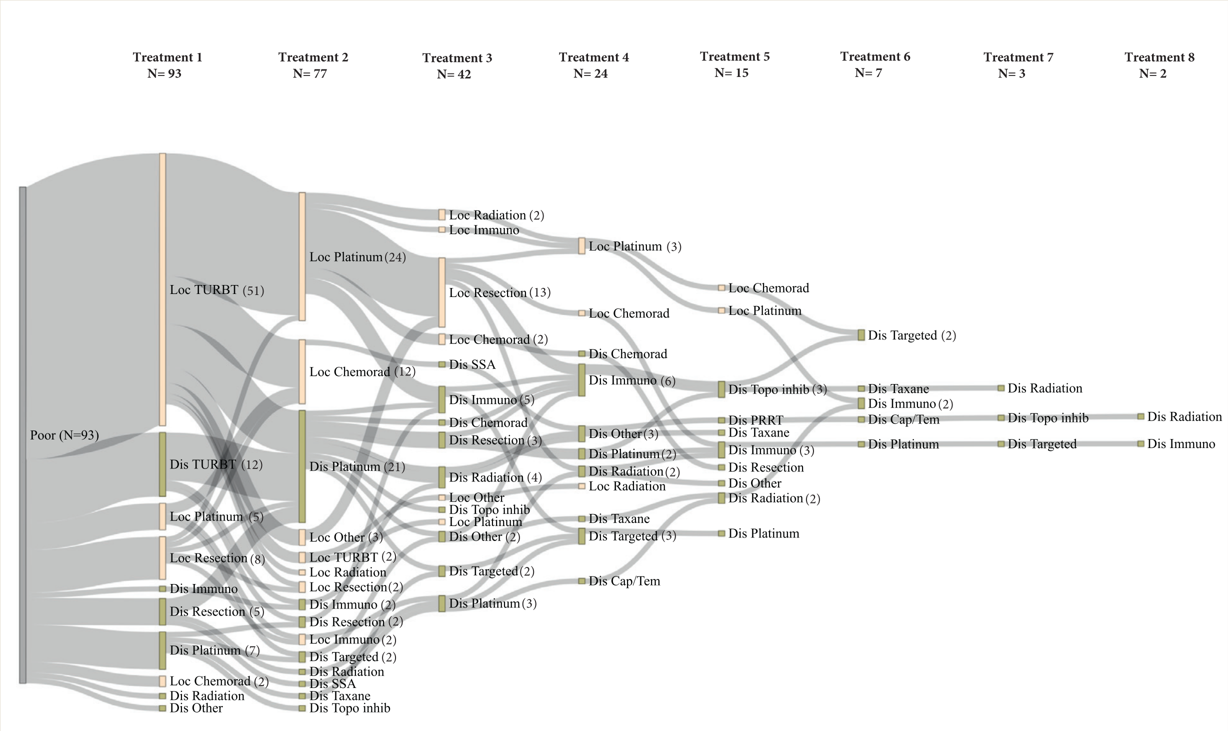

Supplemental Figure 4 Sankey diagrams illustrating treatment sequence from first-line up to tenth-line therapy in WDNETs (N=27). Resection included nephrectomy, hepatic wedge resection. Targeted agents included VEGFR (bevacizumab, cabozantinib, pazopanib, sunitinib), mTORI (everolimus), EGFR (erlotinib) inhibitors. Immunotherapy included PD-1/PD-L1 inhibitors (atezolizumab, nivolumab alone). Oxaliplatin included folfox. 2 PRRTs were combinations: 1 SSA; 1 capecitabine. SSAs were combined in 2 resections, 5 everolimus, 1 cape/tem.

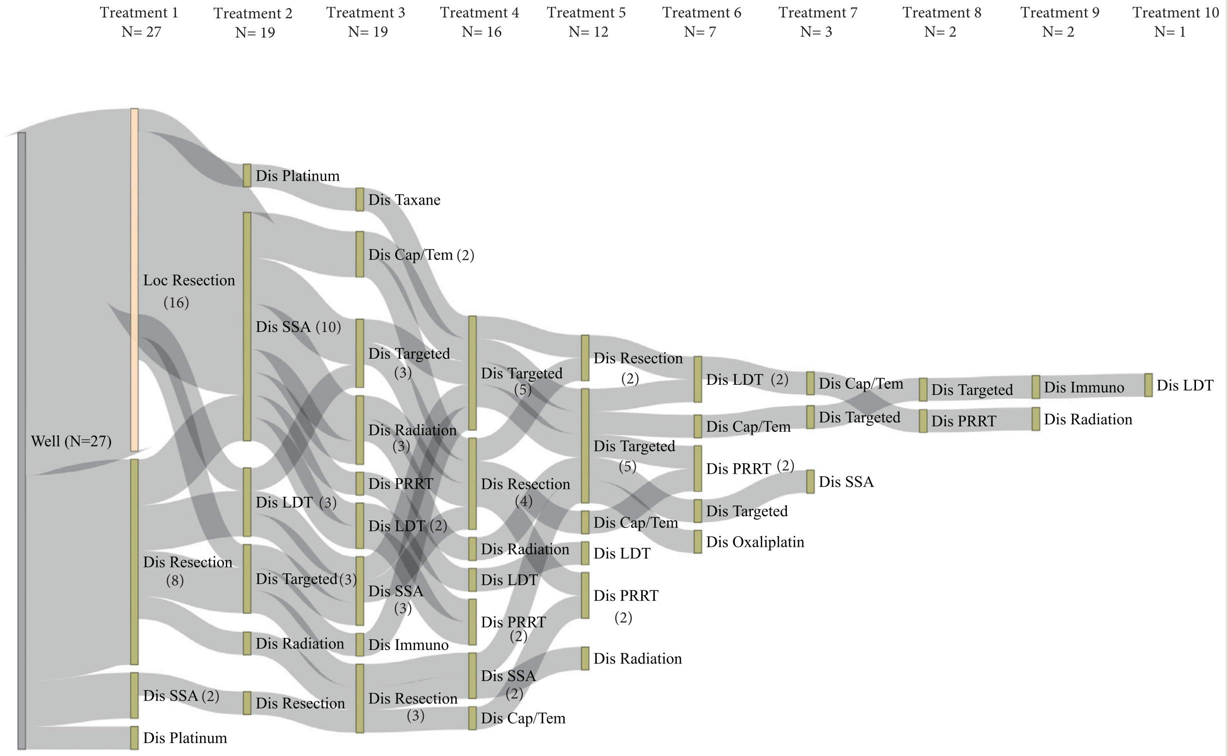

Supplemental Figure 5 Sankey diagrams illustrating treatment sequence from first-line up to tenth-line therapy in Not-reported/ambiguous differentiation (N= 12). Resection included cystectomy, nephrectomy, colectomy, hepatic metastasectomy, lymphadenectomy, brain resection, gastric bypass due to gastric outlet obstruction. Chemoradiation included platinum-based. Targeted agents included VEGFR (pazopanib), mTORI (everolimus). Immunotherapy included PD-1/PD-L1 inhibitors + other (Avelumab + NHS-IL12). Others included ifosfamide + doxorubicin, platinum-based + topoisomerase inhibitors (cisplatin + irinotecan), gemcitabine + ifosfamide + cisplatin, unknown chemotherapy. Oxaliplatin included folfox, oxaliplatin.

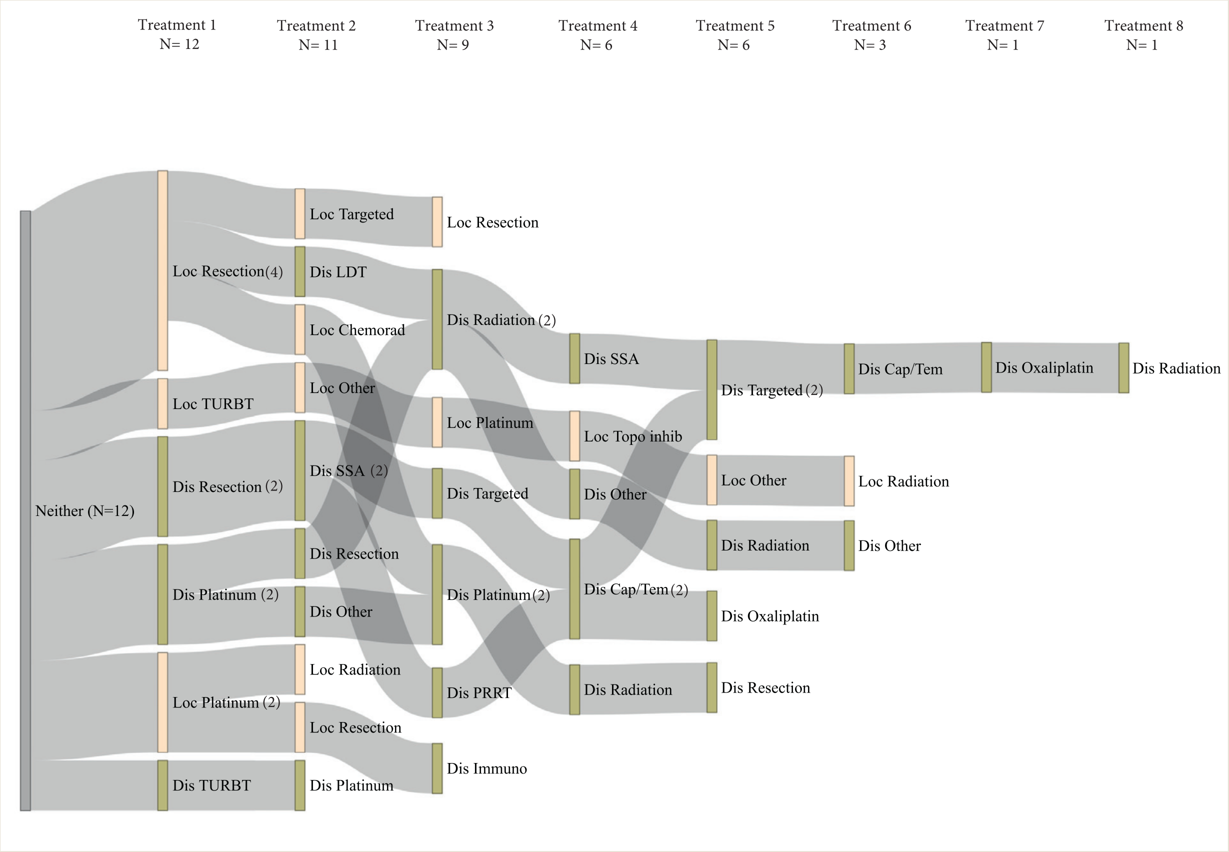

Supplement: 1 [file NIHMS2003043-supplement-1.pdf]
